# Comprehensive considerations for dermatologists: the application of FDG-PET in evaluating cutaneous lesions in pediatric Langerhans cell histiocytosis

**DOI:** 10.3389/fmed.2024.1378638

**Published:** 2024-07-10

**Authors:** Sahithi Talasila, Eric M. Teichner, Robert C. Subtirelu, Naga Chaitanya P. Talasila, Sricharvi Mannam, Thomas Werner, Abass Alavi, Mona-Elisabeth Revheim

**Affiliations:** ^1^Sidney Kimmel Medical College, Thomas Jefferson University, Philadelphia, PA, United States; ^2^Department of Radiology, Hospital of the University of Pennsylvania, Philadelphia, PA, United States; ^3^The Intervention Center, Division of Technology and Innovation, Oslo University Hospital, Oslo, Norway; ^4^Institute of Clinical Medicine, University of Oslo, Oslo, Norway

**Keywords:** Langerhans cell histiocytosis (LCH), pediatric Langerhans cell histiocytosis, cutaneous Langerhans cell histiocytosis, skin-limited Langerhans cell histiocytosis, [18F]fluorodeoxyglucose-positron emission tomography (FDG-PET)

## Abstract

Langerhans cell histiocytosis (LCH) is a complex disorder characterized by the clonal proliferation of Langerhans cells, primarily affecting children and adolescents. This condition exhibits a wide spectrum of clinical presentations, necessitating a multidisciplinary approach for diagnosis, treatment, and follow-up. Cutaneous manifestations of LCH are significant, mimicking common dermatoses and posing diagnostic challenges. [18F]Fluorodeoxyglucose-Positron Emission Tomography (FDG-PET) has emerged as an important tool in the evaluation of pediatric LCH, offering insights into disease activity, extent, and therapeutic response. Moreover, FDG-PET provides a non-invasive means to distinguish between active LCH skin lesions and other dermatological conditions with similar clinical appearances, enhancing diagnostic accuracy and aiding in disease monitoring. This educational review summarizes the utility of nuclear imaging techniques, with a focus on PET scans, in the diagnosis and management of cutaneous pediatric LCH. A comprehensive literature search identified seven relevant articles, including retrospective studies and case reports. These studies highlight the efficacy of FDG-PET in localizing active LCH skin lesions, monitoring disease activity, and guiding treatment decisions. FDG-PET represents a valuable imaging modality for dermatologists, oncologists, and pediatricians managing pediatric LCH patients with cutaneous involvement. This non-invasive technique contributes to improved diagnostic accuracy and facilitates early intervention, ultimately enhancing patient care and outcomes.

## Introduction

Langerhans cell histiocytosis (LCH) is a rare and complex disorder characterized by the clonal proliferation of Langerhans cells, a type of dendritic cell, which normally plays a crucial role in the immune system (1–3). This condition primarily affects children and adolescents, although it can occur in adults as well (4–7). LCH is marked by the infiltration of Langerhans cells into various tissues and organs, leading to a wide spectrum of clinical presentations, ranging from isolated skin lesions to systemic involvement with potentially life-threatening consequences (8–10).

One of the hallmarks of LCH is its unpredictable clinical course, with some patients experiencing spontaneous resolution of lesions, while others endure a chronic and relapsing course that necessitates prolonged treatment and monitoring (11–13). Given its variable presentation and the potential for involvement of virtually any organ system, LCH requires a multidisciplinary approach to diagnosis, treatment, and follow-up (14–16). The disease’s diverse clinical manifestations often pose diagnostic challenges, and its rarity has contributed to limited awareness and research, making it essential to comprehensively understand LCH to improve patient outcomes (17–20).

While virtually any organ can be affected, skeletal involvement stands out as the most prevalent, affecting roughly 90% of pediatric LCH cases (20–23). Additionally, skin manifestations are observed in approximately one-third of patients (20). While its annual incidence is relatively low, approximately 4–4.5 cases per million, LCH’s dermatological presentations impact a significant proportion of patients, making it vital to explore this facet of the disease in detail (24–27). As such, cutaneous manifestations hold particular clinical significance. These skin lesions can take on various forms, including papules, nodules, ulcers, or even chronic eczematous eruptions, often leading to diagnostic challenges due to their resemblance to more common skin conditions (28–30). Given the diverse range of dermatological presentations and their potential for morbidity, understanding and accurately diagnosing skin-specific manifestations are of high importance (31–40). This review aims to summarize the utility of nuclear imaging techniques with emphasis on PET in the diagnosis and treatment of cutaneous pediatric LCH.

## Methods

This educational review was conducted to summarize the utility of nuclear imaging techniques in the diagnosis, management, and follow-up of pediatric LCH. PubMed, Scopus, and ScienceDirect databases were searched for relevant articles discussing the utility of PET scans in pediatric LCH. The search strategy employed a combination of keywords, including “Langerhans cell histiocytosis,” “PET,” “nuclear imaging,” and “pediatric.” A total of 276 citations were identified in the initial search. Two independent reviewers assessed the titles and abstracts of these citations for relevance. Articles meeting the inclusion criteria were selected for full-text review. Studies included those that discussed the use of PET scans in managing pediatric LCH, were published in English language peer-reviewed journals, and were available from January 1985 to September 2023.

## Results

Following the review of titles, abstracts, and duplicates, 225 articles were removed due to being out of scope. Upon further screening, another 22 articles were excluded due to not matching the scope of the study, 20 were excluded due to only including adult patients, one was excluded due to insufficient detail, and one more was excluded due to the full-text not being accessible in English. This left a total of seven articles for analysis (Table 1). Data retrieval encompassed the extraction of pertinent information from each scholarly article, which included key metrics such as patient cohort size, employed imaging modalities, and resultant findings. A systematic narrative synthesis was subsequently executed to encapsulate the efficacy of FDG-PET as a diagnostic and treatment monitoring tool for cutaneous lesions in pediatric LCH. No studies regarding other radiotracers in pediatric LCH were identified in the literature search.

**Table 1:** Demographics and outcomes of the studies included.

Article	Demographics/Subject information	Modality	Summary points	Recommendation on utility (Yes or No)
Teranishi et al. (41)	8-year-old girl with solitary LCH involving the occipital condyle	FDG-PET/CT	In a case report of an 8-year-old girl with Langerhans cell histiocytosis (LCH) involving the occipital condyle, PET/CT imaging played a crucial role in evaluating metabolic activity, revealing neck pain and torticollis as initial symptoms and highlighting the significance of this imaging technique in assessing the extent of disease involvement, particularly in skin lesions, to guide treatment planning and monitor therapeutic response in pediatric LCH cases with diverse organ and tissue manifestations.	Yes
Jorgensen et al. (42)	8-year-old girl presenting with vulvar lesions, found to have multisystem LCH	FDG-PET/CT	In a case report of an 8-year-old girl with Langerhans cell histiocytosis (LCH) featuring vulvar lesions, PET/CT imaging played a crucial role in confirming the diagnosis, assessing the systemic nature of the disease by revealing a concurrent hip lesion, and guiding treatment decisions based on the extent of involvement, underscoring the importance of a comprehensive evaluation and the challenges in diagnosing LCH solely based on cutaneous manifestations.	Yes
Udayasankar et al. (43)	A full-term neonatal boy with congenital multisystem LCH	FDG-PET/CT	In a distinctive case of congenital Langerhans cell histiocytosis (LCH), a neonate with extensive papular skin lesions underwent a comprehensive imaging workup, including contrast-enhanced CT and PET/CT with F-18 FDG, revealing innumerable soft-tissue density nodules and hypermetabolic lymphadenopathy, showcasing the invaluable role of PET/CT in diagnosing, planning treatment, assessing response, and overcoming limitations of conventional imaging in the context of LCH’s heterogeneous clinical presentation.	Yes
Shamim et al. (44)	A 6-year-old with LCH with CNS involvement	FDG-PET/CT	In a case involving a 6-year-old boy with Langerhans cell histiocytosis (LCH) presenting initially with generalized erythematous skin rashes, PET/CT proved instrumental in assessing metabolic activity, aiding in disease activity evaluation and treatment response, highlighting the potential of PET/CT for comprehensive evaluation, early staging, and nuanced management tailored to both cutaneous and systemic manifestations in the multidisciplinary approach to LCH.	Yes
Jessop et al. (45)	33 patients (age 7 weeks to 18 years), of which four had skin-unifocal disease	FDG-PET/CT	A retrospective study analyzing 109 FDG PET-CT scans in 33 pediatric patients with Langerhans cell histiocytosis demonstrates the high sensitivity and specificity of FDG PET-CT in detecting cutaneous lesions, providing valuable insights into its efficacy for comprehensive staging, accurate monitoring of treatment response, and minimizing unnecessary interventions, highlighting its significance in the multidimensional assessment of pediatric LCH beyond cutaneous management.	Yes
Luo et al. (46)	22 patients (age 1–66), of which 14 were pediatric patients	FDG-PET/CT	A retrospective study examining PET/CT images from 22 Langerhans cell histiocytosis (LCH) patients, including 14 pediatric cases, highlights 18F-FDG PET/CT as a valuable tool for diagnosing and evaluating treatment efficacy in LCH, emphasizing its comprehensive view of systemic involvement, including skin lesions, and its ability to provide both anatomical and metabolic information, contributing to a more thorough understanding of disease status beyond conventional imaging methods.	Yes
Niu et al. (47)	15 patients (age 15 months to 9 years)	FDG-PET/CT	In a retrospective analysis of 15 children with Langerhans cell histiocytosis (LCH), the study, although mentioning only two cases with skin manifestations, underscores the potential significance of PET/CT in assessing cutaneous symptoms, emphasizing its established role in skeletal staging, lesion identification, and high sensitivity for detecting metabolic activity, suggesting it as a valuable complementary approach alongside PET/MR, especially in cases with cutaneous involvement.	Yes

In summary, three retrospective studies and four case reports discussed the utility of FDG-PET in pediatric LCH, specifically regarding cutaneous manifestations. The first retrospective study reviewed FDG-PET/CT scans in 33 pediatric patients with LCH, of which four had skin-unifocal disease (41). The second retrospective explored FDG-PET/CT in 22 Chinese pediatric LCH patients (42), while the third retrospective review investigated the use of FDG-PET/MR in 15 pediatric LCH patients (43). The studies highlighted the significant utility of FDG-PET/CT and FDG-PET/MR in managing cutaneous lesions of LCH, offering precise localization and differentiation of active LCH skin lesions from other dermatological conditions, such as benign skin rashes, eczema, and non-inflammatory skin lesions, which are non-FDG avid. The imaging technique plays a crucial role in overall disease evaluation, monitoring treatment response, and guiding therapeutic decisions, and proved to be a tool that dermatologists and oncologists would benefit from understanding in the multidisciplinary care of pediatric LCH patients. Four articles featured case reports on the use of FDG-PET scans in pediatric LCH (44–47), highlighting the use of FDG-PET to monitor disease activity over time and to precisely localize active lesions.

## Discussion

In the realm of pediatric LCH, this compilation of case reports and reviews explores the central role of FDG-PET imaging in diagnosing and managing cutaneous manifestations. Through unique cases of pediatric patients, the multidimensional capabilities of FDG-PET/CT were evident in assessing both cutaneous and systemic aspects of LCH. Retrospective studies emphasize the sensitivity and specificity of FDG-PET, despite radiation exposure concerns, in detecting and monitoring cutaneous lesions. Pediatric dermatologists, as key members of the multidisciplinary team, play a pivotal role in utilizing FDG-PET to diagnose and monitor cutaneous LCH, contributing to tailored treatment plans that address both dermatological and systemic considerations. As we navigate the intricate interplay between cutaneous symptoms and FDG-PET imaging, it becomes clear that a nuanced understanding of this modality is essential for optimizing care and outcomes in pediatric LCH patients.

### Case reports

In a case report of an 8-year-old girl with LCH of the occipital condyle (44), the patient initially presented with neck pain, and PET/CT imaging played a role in evaluating the metabolic activity of the lesion. Although the case report does not extensively detail the cutaneous symptoms, the patient initially presented with neck pain and torticollis. FDG-PET/CT imaging played a crucial role in evaluating the metabolic activity of the lesion, providing insights into the involvement of musculoskeletal and possibly adjacent soft tissue structures, which guided treatment planning and monitoring response to therapy. This multidimensional approach is critical in pediatric LCH cases, where the disease may manifest in various organs and tissues. A similar case report of another 8-year-old female patient highlights the importance of FDG-PET/CT imaging in the diagnosis and management of cutaneous manifestations of pediatric LCH, with a specific focus on vulvar lesions (45). The patient’s vulvar biopsy confirmed the diagnosis of LCH, and subsequent imaging studies, including FDG-PET/CT, revealed a left hip lesion consistent with LCH. This emphasizes the systemic nature of LCH and the need for a comprehensive evaluation to assess the involvement of multiple organ systems. In the context of cutaneous manifestations, PET/CT is particularly valuable for its ability to detect hypermetabolic activity associated with LCH lesions. The report describes a hypermetabolic osteolytic lesion in the left iliac wing, as revealed by FDG-PET/CT, which corresponded to the skeletal radiograph findings. This information is crucial not only for confirming the diagnosis but also for guiding treatment decisions. The systemic chemotherapy (vinblastine, prednisone, and methotrexate) administered to the patient was based on the extent of involvement revealed by imaging studies. Moreover, the case highlights the challenges in diagnosing LCH based solely on the presence of cutaneous lesions, as these may mimic other more common conditions. The report emphasizes the importance of a low threshold for performing biopsies of vulvar lesions, especially in children, to avoid misdiagnosing cutaneous manifestations of diseases that can have significant morbidity. However, less invasive methods such as swabs for HSV or identifying foreign bodies should be considered based on the lesion’s presentation.

In a unique case of congenital LCH, a neonate with extensive papular skin lesions underwent a comprehensive imaging workup, including contrast-enhanced CT and FDG-PET/CT (46). The initial findings revealed innumerable soft-tissue density nodules and hypermetabolic lymphadenopathy in various regions. The use of FDG-PET/CT provided valuable insights into the extent and metabolic activity of the lesions, aiding in both diagnosis and treatment planning. The subsequent follow-up FDG-PET/CT played a crucial role in assessing treatment response by monitoring changes in metabolic activity of the lesions. The follow-up imaging showed a significant reduction in FDG uptake, indicating remission of the disease following systemic chemotherapy. While FDG-PET/CT provided valuable insights into the extent and metabolic activity of the lesions, it is important to note the limitations and barriers associated with this imaging modality in infants. These include the need for sedation or general anesthesia to ensure the infant remains still during the scan, as well as the associated risks and logistical challenges. Additionally, a skeletal survey, which is less invasive and does not require sedation, may be a more appropriate initial imaging modality in some cases. The greatest role of FDG-PET lies in assessing multisystem involvement.

In a case of a 6-year-old boy with LCH, the patient initially presented with generalized erythematous skin rashes (47). In the realm of cutaneous manifestations, FDG-PET/CT provided crucial information about the metabolic activity of lesions, guiding clinicians in assessing disease activity and response to treatment. The case highlights the potential of FDG-PET/CT in offering a holistic evaluation of LCH, including its cutaneous component. This approach not only facilitates early and accurate staging but also contributes to a more nuanced management strategy tailored to the specific manifestations of LCH, both cutaneous and systemic. The findings from this case suggest that FDG-PET/CT can serve as a tool in the multidisciplinary approach to LCH, particularly when cutaneous symptoms are a significant aspect of the clinical presentation.

All case reports presented highlight the crucial role of FDG-PET in diagnosing and managing cutaneous manifestations of pediatric LCH. The cases highlight the systemic nature of LCH, emphasizing the need for a holistic evaluation to assess the involvement of multiple organ systems. FDG-PET/CT imaging were found to be a valuable tool in evaluating the metabolic activity of lesions, aiding in both diagnosis and treatment planning. FDG-PET/CT proved essential in cases where cutaneous symptoms were significant, offering insights into the distribution and severity of cutaneous involvement.

### Comprehensive reviews

One retrospective study investigated 109 FDG-PET/CT scans in 33 pediatric patients, offering valuable insights into the use of this imaging modality for cutaneous symptoms (41). FDG-PET/CT proves highly sensitive, allowing for comprehensive staging and effective follow-up during treatment. The study demonstrated that FDG-PET/CT was adept at detecting all types of LCH lesions, including cutaneous lesion, and assessing the extent of disease involvement. Particularly in the context of LCH, where skin involvement is observed in approximately 33% of cases, the sensitivity of FDG-PET/CT becomes important. The ability to identify metabolic activity in the skin lesions facilitates accurate staging and monitoring of treatment response. Moreover, the low per-patient false-positive rate of 4% found at staging indicated the reliability of FDG-PET/CT in minimizing unnecessary interventions. The study’s emphasis on the specificity and sensitivity of FDG-PET/CT during follow-up, with a per-scan false-positive rate of 2%, highlighted its efficacy in tracking disease recurrence and progression specifically in cutaneous LCH. While acknowledging the inevitable increase in radiation exposure associated with FDG-PET/CT, the study contended that the benefits of accurate staging, monitoring, and detection of cutaneous lesions outweigh the potential risks. This is particularly relevant in cases where conventional imaging modalities may fall short in providing a comprehensive assessment of cutaneous symptoms. However, with the whole body PET systems and PET/MRI, the radiation exposure is significantly reduced. The final conclusions of the study do not make a recommendation as to the utility of FDG-PET/CT as a valuable tool in solely the cutaneous management of pediatric LCH, but encourage the use of this imaging modality in the comprehensive assessment of pediatric LCH.

Another retrospective study analyzed FDG-PET/CT images from 22 patients, of which 14 were pediatric, diagnosed with LCH, and revealed that FDG-PET/CT was a valuable tool for diagnosing and evaluating treatment progress in LCH, extending its significance beyond conventional imaging methods (42). Skin and soft tissue involvement accounted for 22.7% of cases. FDG-PET/CT, as a whole-body imaging technique, offered a comprehensive view of systemic LCH involvement, including cutaneous lesions. This is crucial in the context of LCH, where lesions can affect multiple sites and may not always be apparent through conventional imaging alone. Moreover, the study noted that FDG-PET/CT provides both anatomical details and metabolic information about lesions. In the context of cutaneous symptoms, this dual capability is particularly relevant. While conventional imaging methods like X-ray, CT, and MRI play key roles in diagnosing and evaluating the extent of LCH, FDG-PET/CT adds a metabolic dimension. This is essential for assessing the activity of lesions, including those in the skin, and can contribute to a more comprehensive understanding of disease status. While the study touched on skin involvement in pediatric LCH, it did not provide an in-depth analysis of cutaneous manifestations. However, it highlighted the significance of FDG-PET/CT in the overall management of LCH, emphasizing its potential in evaluating cutaneous symptoms along with other organ involvement.

A final retrospective analysis studied 15 children with LCH. However, regarding the cutaneous aspect, the study mentions only two cases with skin manifestations (43). Despite the limited mention, it is crucial to discuss the implications of PET/MR and PET/CT in assessing cutaneous symptoms. PET/CT, with its sensitivity to skeletal staging and ability to identify lesions smaller than 10 mm, has been established as a significant tool in the diagnosis and evaluation of pediatric LCH. While FDG-PET/MR is highlighted for its reduced radiation exposure, FDG-PET/CT remains a valuable tool with high sensitivity for skeletal and soft tissue lesions, making it a potential complementary approach in the comprehensive evaluation of cutaneous symptoms in pediatric LCH.

The retrospective studies provided valuable insights into the use of FDG-PET/CT in managing cutaneous lesions of LCH. FDG-PET/CT’s high sensitivity is emphasized, allowing for comprehensive staging and effective follow-up during treatment. The specificity and sensitivity of FDG-PET/CT during follow-up highlight its efficacy in tracking disease recurrence and progression, which can also be clinically correlated in cutaneous LCH. The articles do not explicitly recommend FDG-PET/CT as a sole tool for cutaneous management but encourage its use in the comprehensive assessment of pediatric LCH, considering its high sensitivity, specificity, and ability to offer a whole-body perspective.

**Figure 1:**
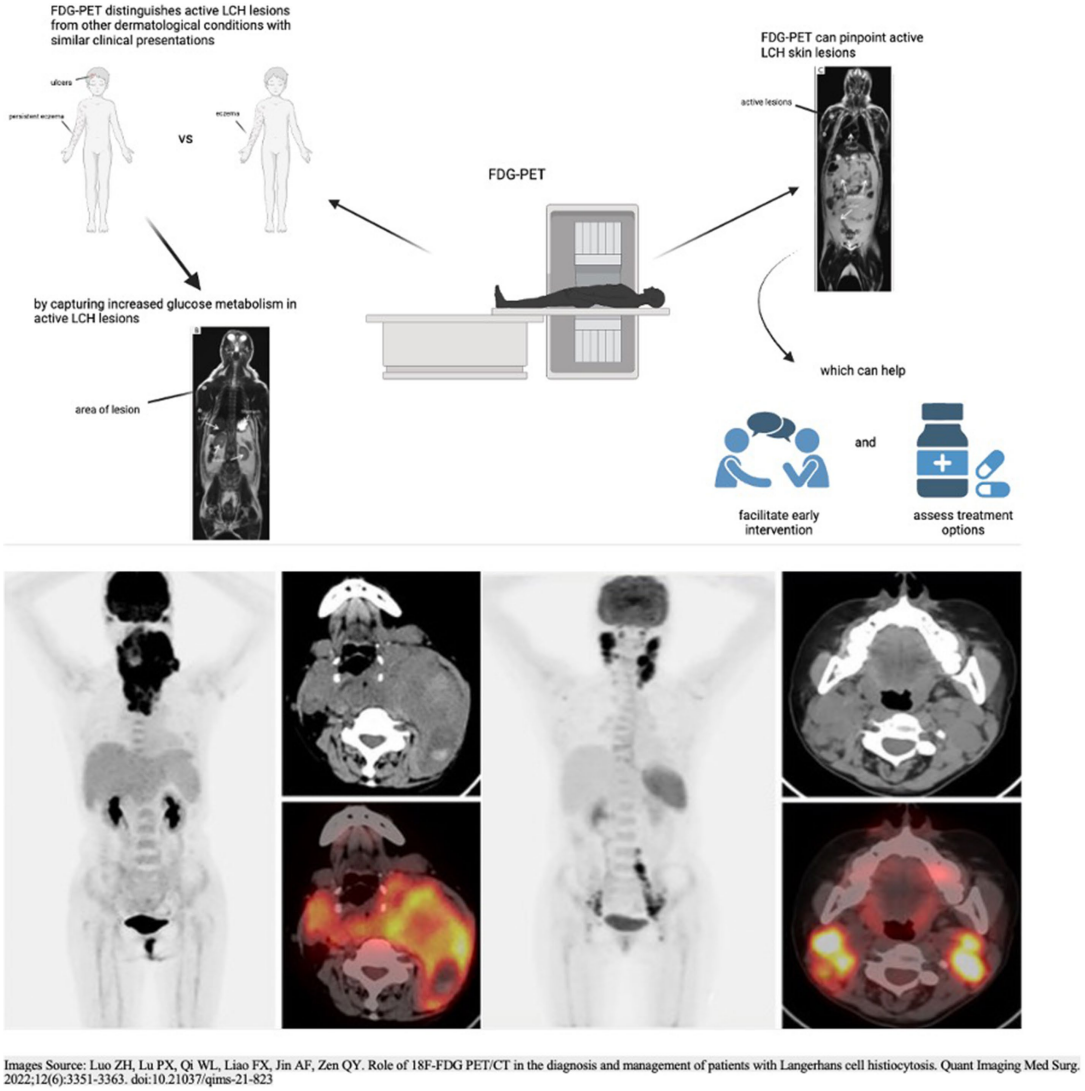
Utility and applications of FDG-PET in the distinction and detection of LCH lesions. The two PET scans in the top half of the figure and the PET scans in the bottom half of the figure: Adapted with permission from Luo et al. (46), licensed under CC BY-NC-ND 4.0, https://doi.org/10.21037/qims-21-823.

Pediatric dermatologists play a pivotal role in the multidisciplinary care of pediatric Langerhans cell histiocytosis patients. Given the complex and variable nature of LCH, including its cutaneous manifestations, pediatric dermatologists should become familiar with the use and interpretation of FDG-PET scans to enhance their ability to contribute effectively to the multidisciplinary team. Pediatric dermatologists are often the first point of contact for patients presenting initially with cutaneous symptoms (48). FDG-PET provides a means to distinguish between active LCH skin lesions and other dermatological conditions with similar clinical appearances. By capturing increased glucose metabolism, FDG-PET enables precise localization and differentiation of LCH skin lesions. This capability is crucial for accurate diagnosis, especially when cutaneous manifestations mimic common skin conditions, allowing pediatric dermatologists to initiate appropriate and timely interventions (Figure 1).

Understanding the systemic nature of LCH is essential for effective management. Pediatric dermatologists, in collaboration with other specialists, can utilize FDG-PET to assess the extent of disease involvement beyond the skin. This comprehensive, whole-body assessment aids in the identification of both common and rare sites of involvement (49).

In the multidisciplinary care of pediatric LCH patients, treatment decisions are often guided by the extent of disease involvement. The ability of FDG-PET to monitor treatment response over time allows pediatric oncologists and other specialists to actively participate in adjusting therapeutic strategies. This real-time feedback enhances the collaborative efforts of the multidisciplinary team, ensuring that interventions are tailored to the evolving needs of the patient. Effective management of LCH requires close collaboration among pediatric oncologists, dermatologists, radiologists, and nuclear medicine physicians. Understanding and interpreting FDG-PET scans enable effective communication within the multidisciplinary team, fostering a collaborative approach to diagnosis, treatment, and follow-up. By actively participating in discussions related to PET findings, each specialist contributes unique insights into different aspects of the disease, enriching the overall understanding of the patient’s condition (50, 51).

However, one significant limitation of FDG-PET imaging is the potential for false-positive and false-negative results (51). False-positive results can occur due to the non-specific nature of FDG uptake, which can be seen in various inflammatory and infectious conditions that mimic LCH. On the other hand, false-negative results might occur in lesions with low metabolic activity or in small lesions below the resolution threshold of the PET scanner. These inaccuracies can hinder the effective diagnosis and monitoring of LCH, potentially delaying appropriate treatment interventions (51).

Radiation exposure is also a critical concern, especially in pediatric patients. Children are more sensitive to radiation, and the cumulative exposure from multiple imaging studies can increase the risk of radiation-induced malignancies later in life. Advances in imaging technology, such as FDG-PET/MRI, offer reduced radiation exposure but are not yet universally available or standardized in clinical practice (48, 49).

[18F]Fluorodeoxyglucose-Positron Emission Tomography imaging also has technical limitations that can impact its effectiveness. Spatial resolution, although improving, can still be insufficient for detecting very small lesions, which is particularly relevant in cutaneous LCH where lesions can be small and widespread. Additionally, the interpretation of FDG-PET images requires significant expertise to diagnose LCH.

One significant limitation of FDG-PET imaging is the requirement for general anesthesia or sedation in pediatric patients to ensure they remain still during the scan. This carries associated risks, such as potential complications from anesthesia and the need for specialized medical personnel and facilities, which may not always be available. Additionally, the availability of FDG-PET/CT is limited in some settings, particularly in low-and middle-income countries, due to high costs, the need for advanced technology, and the infrastructure required to maintain and operate the equipment (52).

## Conclusion

Our review supports FDG-PET as an important diagnostic and monitoring tool for cutaneous lesions in pediatric LCH. It is particularly valuable in cases with significant cutaneous involvement, offering precise localization, differentiation of active LCH skin lesions from other dermatological conditions, and monitoring metabolic changes over time. A multidisciplinary approach, involving dermatologists, oncologists, and pediatricians, is crucial in the comprehensive management of pediatric LCH, with FDG-PET playing a significant role in guiding therapeutic decisions and improving patient care and outcomes.

## Author contributions

ST: Conceptualization, Data curation, Formal Analysis, Funding acquisition, Investigation, Methodology, Project administration, Resources, Software, Supervision, Validation, Visualization, Writing – original draft, Writing – review & editing. ET: Conceptualization, Data curation, Formal Analysis, Funding acquisition, Investigation, Methodology, Project administration, Resources, Software, Supervision, Validation, Visualization, Writing – original draft, Writing – review & editing. RS: Conceptualization, Data curation, Formal Analysis, Funding acquisition, Investigation, Methodology, Project administration, Resources, Software, Supervision, Validation, Visualization, Writing – original draft, Writing – review & editing. NT: Conceptualization, Data curation, Formal Analysis, Funding acquisition, Investigation, Methodology, Project administration, Resources, Software, Supervision, Validation, Visualization, Writing – review & editing. SM: Conceptualization, Data curation, Formal Analysis, Funding acquisition, Investigation, Methodology, Project administration, Resources, Software, Supervision, Validation, Visualization, Writing – review & editing. TW: Conceptualization, Data curation, Formal Analysis, Funding acquisition, Investigation, Methodology, Project administration, Resources, Software, Supervision, Validation, Visualization, Writing – review & editing. AA: Conceptualization, Data curation, Formal Analysis, Funding acquisition, Investigation, Methodology, Project administration, Resources, Software, Supervision, Validation, Visualization, Writing – review & editing. M-ER: Conceptualization, Data curation, Formal Analysis, Funding acquisition, Investigation, Methodology, Project administration, Resources, Software, Supervision, Validation, Visualization, Writing – original draft, Writing – review & editing.
